# Monotropein inhibits epithelial–mesenchymal transition in chronic colitis via the mTOR/P70S6K pathway

**DOI:** 10.3389/fphar.2025.1536091

**Published:** 2025-02-18

**Authors:** Yuanfan Chen, Jiaying Liu, Shaowen Zhong, Tianwu Zhang, Jin Yuan, Jing Zhang, Ying Chen, Jian Liang, Yonger Chen, Shaozhen Hou, Haiyang Huang, Jie Gao

**Affiliations:** ^1^ School of Pharmaceutical Sciences, Guangzhou University of Chinese Medicine, Guangzhou, Guangdong, China; ^2^ College of Education, Guangzhou Huali Science and Technology Vocational College, Guangzhou, Guangdong, China; ^3^ College of Education, University of Visayas, Cebu, Philippines; ^4^ Pu’er Hospital of Traditional Chinese Medicine, Puer, Kunming, Yunnan, China; ^5^ School of Basic Medical Sciences, State Key Laboratory of Respiratory Disease, Sino-French Hoffmann Institute, Guangzhou Medical University, Guangzhou, Guangdong, China; ^6^ Development Planning Department, Guangzhou University of Chinese Medicine, Guangzhou, Guangdong, China

**Keywords:** monotropein, DSS, epithelial–mesenchymal transition, mTOR/P70S6K, chronic colitis

## Abstract

**Introduction:**

Patients with chronic colitis are at risk of developing intestinal fibrosis through epithelial–mesenchymal transition (EMT). Monotropein (MON) is the main active ingredient in the traditional Chinese medicine *Morinda officinalis How*. It has been reported that monotropein can improve ulcerative colitis, but the mechanism remains unclear. However, whether monotropein can improve chronic colitis-associated intestinal fibrosis remains unknown. The study aimed to investigate the effect of monotropein on EMT in chronic colitis and its underlying mechanism.

**Methods:**

The mice chronic colitis model was induced by dextran sodium sulfate (DSS). Cytokines were detected by ELISA. Concentrations of fluorescein isothiocyanate dextran (FITC-Dextran) in serum were detected using a fluorescein microplate analyzer. Intestinal tight junction proteins were detected by immunofluorescence. EMT marker proteins were detected by immunohistochemistry. Transforming growth factor-β1 (TGF-β1) was used to induce EMT in IEC-6 cells. Western blot, real-time quantitative PCR, and immunofluorescence were used to test the inhibitory effect of monotropein on the development of EMT and explore its mechanism.

**Results:**

Results showed that monotropein significantly improved colonic injury and inhibited the expression of colonic tissue EMT marker protein. In addition, molecular docking and molecular dynamics (MD) simulation, cellular thermal shift assay (CETSA), and drug affinity responsive target stability (DARTS) assay validated monotropein targeting of mTOR. Monotropein inhibited TGF-β1-induced EMT in IEC-6 cells, inhibited the phosphorylation of mTOR and its downstream proteins, and increased the autophagy activity in chronic colitis mice and IEC-6 cells.

**Discussion:**

The study indicates that monotropein inhibits the development of EMT in DSS-induced chronic colitis mice and TGF-β1-induced IEC-6 cells. Its inhibitory effect on EMT is associated with the mTOR/P70S6K pathway.

## Graphical Abstract

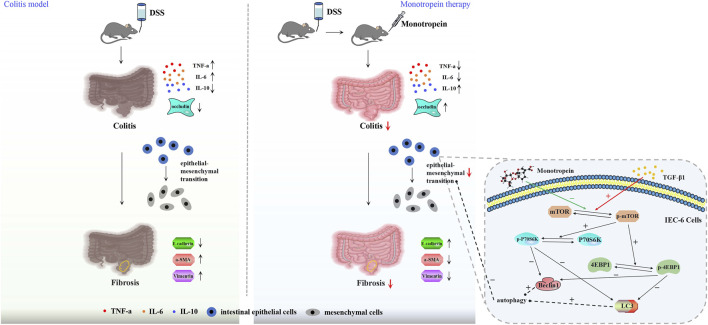

## 1 Introduction

Inflammatory bowel disease (IBD) is a recurrent inflammatory disorder of unknown etiology, primarily driven by abnormal immune responses, and can affect the entire digestive tract and extra-intestinal organs. The global incidence of IBD is on the rise and primarily encompasses ulcerative colitis (UC) and Crohn’s disease (CD) (Barreiro-de Acosta et al., 2023). Many studies supported the role of inflammation in the induction of intestinal fibrosis in Crohn’s disease (Torle et al., 2020; Macias-Ceja et al., 2019). While acute intestinal inflammation is typically followed by tissue repair and restoration of intestinal structure and function under normal physiological conditions, chronic inflammation in the intestine is characterized by repetitive tissue damage and repair cycles, ultimately leading to the onset and progression of intestinal fibrosis. Chronic inflammation also fuels cancer development via the epithelial–mesenchymal transition (EMT), a process where epithelial cells transform into active mesenchymal cells (Flier et al., 2010; Scharl et al., 2015). Therefore, the inhibition of EMT may be a key process in preventing the transformation of colitis into fibrosis and cancer.

The occurrence of EMT is related to a variety of cytokines, protein molecules, microenvironment, and microRNA (Dominguez et al., 2017; Suarez-Carmona et al., 2017; Solinas et al., 2010; Lopez-Novoa and Nieto, 2009; Zou et al., 2017), which involves a large cell signal transduction pathway and complex gene regulation process. Transforming growth factor-β (TGF-β) is the most important fibrogenic factor and an important molecule in the process of inducing EMT (Shi et al., 2019; Tian et al., 2017; Sferra et al., 2018; Yun et al., 2019). The downregulation of epithelial cell markers such as E-cadherin and the upregulation of mesenchymal cell markers like α-smooth muscle actin (α-SMA) and vimentin serve as significant indicators of EMT (Lamouille et al., 2014).

Autophagy is one of the main mechanisms that inhibit EMT (Nieto et al., 2016). The expression of autophagy-related gene (ATG) inhibits EMT (Jo et al., 2017), while the Beclin 1 or ATG7 gene knockout increases the expression of the EMT regulatory factor Snail (Grassi et al., 2015). Mammalian target of rapamycin (mTOR), a critical protein involved in autophagy regulation, is a central target in this process. Studies have reported that mTOR inhibitors or mTOR knockout can suppress the expression of IL-23 in mononuclear phagocytes, decrease the expression of IL-22 in mouse intestinal fibrosis models, and ameliorate mouse intestinal fibrosis, effects associated with increased autophagy activity (Mathur et al., 2019).

Monotropein (MON) is the main active ingredient in the traditional Chinese medicine *Morinda officinalis How*, which has been shown to improve ulcerative colitis, but the mechanism remains unclear (Shin et al., 2013a). In traditional Chinese medicine, *Morinda officinalis* is often used to nourish the kidneys, strengthen the bones, and improve overall vitality. Monotropein, as its main active component, has been reported to possess anti-inflammatory, antioxidant, and immune-modulatory properties (Shin et al., 2013b).

Previous studies have shown that monotropein can improve ulcerative colitis, but the mechanism remains unclear. Additionally, monotropein has been reported to regulate autophagy through the Akt/mTOR pathway in other contexts (Etemadi et al., 2020). Autophagy is a critical cellular process involved in the degradation and recycling of damaged or excess proteins and organelles, and it plays a pivotal role in maintaining cellular homeostasis (Zhu et al., 2024). Importantly, autophagy has been implicated in the regulation of EMT, with increased autophagy activity being associated with the inhibition of EMT (Gundamaraju et al., 2022b). Based on these findings, we speculate that monotropein might inhibit EMT by regulating autophagy via the mTOR/P70S6K pathway.

The aim of this experiment is to investigate the effect of monotropein on EMT in chronic colitis and its underlying mechanism. A chronic colitis mice model was established with 2%DSS and administered with monotropein to study its improving effect on fibrosis through inhibiting EMT and the mechanism. IEC-6 cells were induced with TGF-β1 to establish an *in vitro* fibrosis model to study the role of the mTOR/P70S6K signaling pathway for the effect of monotropein on EMT. This study might offer some reference for applying monotropein and herbs rich in monotropein to improve IBD and prevent IBD from transforming into fibrosis.

## 2 Materials and methods

### 2.1 Materials

**FIGURE 1:**
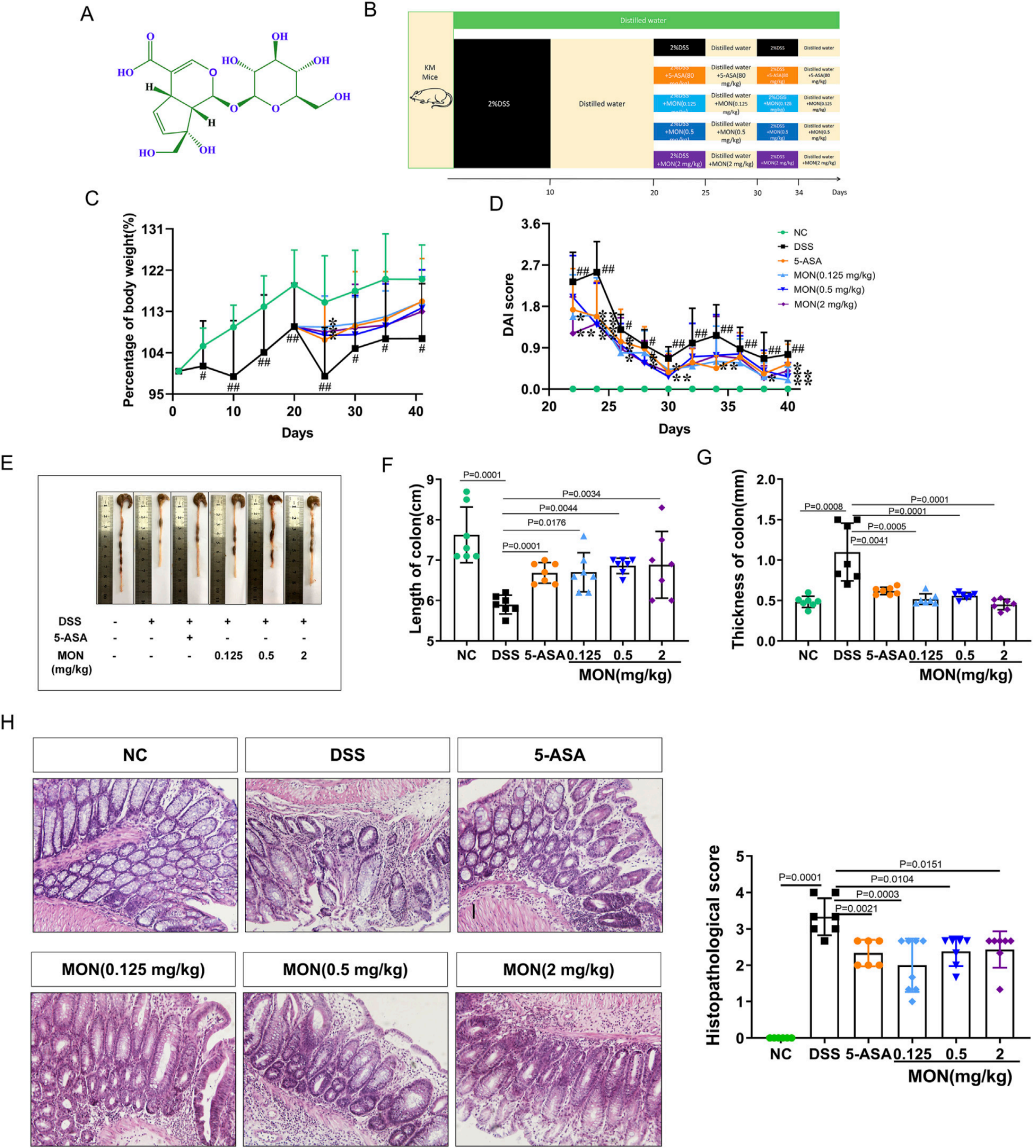
Monotropein alleviates colonic injury in a chronic colitis mice model. **(A)** The structure of monotropein. **(B)** Schematic diagram of molding and dosing cycles in animal experiments. **(C)** Percentage of body weight (%). **(D)** DAI score. **(E)** Representative colon images. **(F)** Length of colon (cm). **(G)** Thickness of colon (mm). **(H)** Left: Histopathological score. Right: Representative pictures of H&E of each group (*n* = 3). The results are presented as mean ± SD (*n* = 8). ^#^ *P* < 0.05, ^##^ *P* < 0.01. Compared to the model group, ^*^ *P* < 0.05, ^**^ *P* < 0.01.

Monotropein (CAS# [5945-50-6], HPLC 98%) was purchased from Baoji Herbest Bio-Tech Co., Ltd. (China). Molecular formula: C_16_H_22_O_11_. The structure of the compound is shown in Figure 1A.

### 2.2 Animal

Male Kunming (KM) mice were obtained from the Laboratory Animal Center of Guangzhou University of Traditional Chinese Medicine (License No. SCXK 2013-0002). All experimental mice were bred in specific pathogen-free conditions of 20 ∼ 25°C temperature, 55% ± 10% humidity, and 12-h/12-h light/dark cycle with 24-h free access to standard feed and sterilized water. The experimental scheme was permitted by the Animal Ethics Committee of Guangzhou University of Chinese Medicine (License No. 20210824012) and was conducted in accordance with the US National Institutes of Health guidelines for humane animal use (NIH Publications, No. 8023, revised 1978).

### 2.3 Establishment and treatment of experimental chronic colitis

Dextran sodium sulfate (DSS, MW 36,000–50,000) was purchased from HuicH (China). After 1 week of animal adaptation feeding, the weighted random sampling method was used for grouping. Specifically, six containers were prepared, each containing an equal number of balls numbered from 1 to the total number of animals. The animals are weighed in turn, and a corresponding number of pellets are drawn from the container according to their weight. The extracted numbers were used to assign the animals to six groups: one normal group and five model groups (Gellerstedt, 2002). One group was randomly selected as the normal group, and the remaining five groups of animals were remixed to establish a chronic colitis model.

The model building was divided into three cycles. In the first cycle, all animals in the modeling group were given 2% DSS for 10 days, followed by sterilized water for 10 days. In the second cycle, animals in the DSS group were given 2%DSS for 5 days, followed by sterilized water for 5 days. In the third cycle, animals in the DSS group were given 2%DSS for 4 days, followed by sterilized water for 4 days. In all cycles, normal group animals were given sterilized water every day. The dosage of monotropein was referred to in a previous report (Chen et al., 2020) and was combined with the effective dose of the pre-experiment. Monotropein was dissolved in sterilized water. Administration began with the second cycle. After the first cycle of modeling, the modeling group was randomly divided into a DSS group, a 5-ASA (80 mg/kg) group, a monotropein low-dose (0.125 mg/kg) group, a monotropein medium-dose (0.5 mg/kg) group, and a monotropein high-dose (2 mg/kg) group according to the weight random principle (*n* = 11). The 5-ASA and monotropein treatments were administered daily throughout the second and third cycles (18 days). The DSS group received DSS according to the schedule mentioned above. From the second cycle to the end of the third cycle, 0.1 mL/10 g of the drug was given daily by intragastric administration (Figure 1B). The animals’ weights were recorded daily. At the end of modeling and administration, the animals were anesthetized with pentobarbital sodium (40 mg/kg), blood was collected, and the colon tissues were separated.

### 2.4 Disease activity index

The disease activity index (DAI) was determined by referring to the method of Tohru (Funakoshi et al., 2012) and our previously described method (Zhang et al., 2022).

### 2.5 Histological evaluation

Colon tissues were made into tissue slices (4 μm) and stained using an H&E kit (BKMAM, China). The extent of colon tissue injury was observed under a microscope (BX53, Japan). The scoring for colon injury was performed according to the previous studies (Zhang et al., 2022; Zhou et al., 2019).

### 2.6 Detection of cytokines

At the end of the third model-building cycle, animal serum was collected for the detection of cytokines. Animal serum was separated at 4°C and centrifuged at 3500 r/min for 15 min. The levels of various cytokines (TNF-a, IL-6, and IL-10, Meimian Biotechnology, China) were detected in the serum according to the ELISA kit instructions.

### 2.7 Permeability of FITC-dextran

The mice were fasted for 12 h and given FITC-dextran (50 mg/kg) (MedChemExpress, United States). Four hours later, peripheral blood was collected and left in the dark for 1 h. Animal serum was separated at 4°C and centrifuged at 3,500 r/min for 15 min. Serum fluorescence intensity was measured, and the concentration of FITC-dextran in serum was calculated by a standard curve.

### 2.8 Immunofluorescence assay

Slices were dewaxed with xylene and hydrated in different concentrations of alcohol for 5 min. The slices were used to repair antigens in citric acid repair solution and blocked with BSA (0.1%) for 30 min. The tissues were incubated with a suitable concentration of primary antibodies (Table 1) at 4°C overnight and then incubated with secondary antibody for 2 h in the dark. Finally, DAPI was added to the tissue and incubated for 5 min against light, and the slices were observed under a confocal Zeiss microscope (Carl Zeiss, Germany).

**TABLE 1:** Antibody information.

Antibody		Dilution rate	Manufacturer	Cat. #
Primary antibody	occludin	1:200	Affinity	DF7504
	ZO-1	1:200	Affinity	AF5145
	E-cadherin	1:200/1:1,000	Affinity	AF0131
	Vimentin	1:1,000	Signalway antibody	33541
	a-SMA	1:1,000	Proteintech	55135-1-AP
	mTOR	1:1,000	Signalway antibody	41187
	p-mTOR	1:1,000	Cell signaling	2974P
	p70S6K	1:1,000	Affinity	AF6226
	p-p70S6K	1:1,000	Affinity	AF3228
	4EBP1	1:1,000	Signalway antibody	33127
	p-4EBP1	1:1,000	Signalway antibody	11222
	Beclin1	1:200/1:1,000	Affinity	AF5128
	LC3	1:1,000	Affinity	AF5402
	GAPDH	1:4,000	Affinity	AF7021
Secondary antibody	Goat Anti-Rabbit lgG (H + L) HRP	1:4,000	Affinity	S0001

IEC-6 cells were treated with relevant reagents. Then, the corresponding primary antibody (E-cadherin, Beclin1) and fluorescently coupled secondary antibody were incubated for immunofluorescence staining. Nuclei were stained with DAPI. A confocal Zeiss microscope (Carl Zeiss, Germany) was used to examine and photograph cells in a random field of view.

### 2.9 Sirius red staining and Masson’s trichrome assay

The experiment was carried out following our previously described method (Xu et al., 2024; Chen Y.-E. et al., 2021).

### 2.10 Immunohistochemistry assay

The slices were dewaxed to water and were used to repair antigens in a citric acid repair solution. The tissues were washed two times, 3 min each time with PBS, and then were incubated with hydrogen peroxide block for 15 min. The tissues were washed with PBS two times, 5 min each time, and blocked with BSA (3%) for 15 min. The tissues were incubated with a-SMA antibody at 37°C for 2 h. The tissues were washed with PBS three times, 3 min each time, and incubated with HRP polymer at room temperature for 20 min. The tissues were incubated with *streptomyces* vitellin working solution labeled with horseradase at room temperature for 20 min. The tissues were washed with PBS three times, 3 min each time, and DAB was used to develop color for 30 s. The tissues were rinsed with running water, dehydrated, sealed with neutral gum, and finally observed under a microscope (BX53, Japan).

### 2.11 Cell culture and treatment

Rat small intestine crypt epithelial cells (IEC-6) were cultured in DMEM (Gibco, United States) containing 10% fetal bovine serum (Gibco, United States) and a 1% mixture of 100 U/mL streptomycin and penicillin (Gibco, United States) in a 5% CO_2_ incubator at 37°C.

IEC-6 cells were implanted into six-well plates at a density of 3 × 10^5^/well and divided into five groups (n = 3): control group, TGF-β1-treated group (TGF-β1), and three monotropein-treated TGF-β1 groups (MON + TGF-β1 (5 µM, 10 µM, and 20 µM)). We used TGF-β1 (PeproTech, China) to induce EMT in IEC-6. The dosage of monotropein referred to the result of the cell viability test experiment and was combined with the effective dose of the pre-experiment. When the cells grew to 30%, they were cultured with TGF-β1 (10 ng/mL) to induce EMT for 72 h. During the establishment of the EMT model, cells were cultured with DMEM with 1% fetal bovine serum and a 1% mixture of 100 U/mL streptomycin and penicillin, along with gradient concentrations of monotropein, for 72 h.

### 2.12 Cell viability

A cell counting kit-8 (CCK-8; GLPBIO, United States) assay was used to measure cell viability. The experiment followed our previously described method (Lu et al., 2022).

### 2.13 Western blot

In order to detect the expression levels of proteins related to EMT and the mTOR/P70S6K pathway, the colonic tissue and IEC-6 cells were processed by the general Western blot steps. The equivalent proteins were separated with a 10% SDS-polyacrylamide gel and then transferred to a polyvinylidene fluoride (PVDF) membrane. The membranes were cut horizontally at the appropriate location according to the molecular weight of the target proteins. Next, the membranes were blocked for 2 h and incubated with a suitable concentration of primary and secondary antibodies (Table 1). Finally, bands were washed with TBST and examined by ECL (Millipore, United States). The intensities of bands were calculated by ImageJ.

### 2.14 Real-time quantitative PCR

The total RNA of cells was extracted with the RNAex Pro reagent (Accurate Biotechnology, China). Next, an Evo M-MLV RT Kit with gDNA Clean for qPCR II (Accurate Biotechnology, China) was used to reverse-transcribe RNA samples to cDNA. The cDNA was amplified with the SYBR Green Premix Pro Taq HS qPCR Kit (Accurate Biotechnology, China) and target gene primers (Table 2) in a detection system (CFX96 TouchTM, Bio-Rad, United States). The reaction condition was as follows: 95°C for 30 s, 40 cycles of 95°C for 5 s, and 60°C for 30 s. Quantification was conducted using the 2^−△△Ct^ method. All target genes were normalized with GAPDH (Sangon Biotech, China).

**TABLE 2:** mRNA primers of the IEC-6 cell.

mRNA (gene ID)	Forward	Reverse
E-cadherin (83502)	TGAAGAGGGAGGTGGAGA	GACGGGGACGATACTGG
Vimentin (81818)	AGGTGGAGAGGGACAACC	GGTCAAGACGTGCCAGAG
a-SMA (81633)	CCG​CAA​ATG​CTT​CTA​AGT​C	GCGCTGATCCACAAAAC
Beclin1 (114558)	GGC​CAG​ACA​GTG​TTG​TTG​CT	CCC​CAG​AAC​AGT​ACA​ACG​GC
LC3 (362245)	GCAGCAGATCCGTGACC	GCT​TCT​CAC​CCT​TGT​AGC​G

### 2.15 Cellular thermal shift assay (CETSA)

The CETSA experiment followed our previously described method (Chen et al., 2024; Liu et al., 2024).

### 2.16 Drug affinity responsive target stability (DARTS) assay

The DARTS assay followed our previously described method (Chen et al., 2024).

### 2.17 Molecular docking and molecular dynamics (MD) simulation

Molecular docking was performed with AutoDock Vina 1.0.2. Molecular docking and kinetic simulations were performed as described in previous articles (Chen et al., 2024; Chen et al., 2022).

### 2.18 Statistical analysis

All data were expressed as mean ± standard deviation (SD). The differences within the group comparison were analyzed by a one-way ANOVA test followed by Tukey’s test. *P* < 0.05 was considered to indicate significant differences.

## 3 Results

### 3.1 Monotropein could alleviate colonic injury in the chronic colitis mice model

To investigate the impact of monotropein on chronic colitis, a mouse model of the disease was established using 2% DSS, with 5-ASA or monotropein administered during the second and third modeling cycles (Figure 1B). During the second cycle of modeling, the DSS-treated group exhibited significant weight loss compared with the control group (Figure 1C). However, both 5-ASA and various doses of monotropein significantly mitigated this weight loss when compared to the model group (Figure 1C). In both the second and third cycles, the DSS group experienced severely loose and bloody stools, with a marked increase in DAI scores compared to the control group (Figure 1D). Conversely, the mice treated with 5-ASA or monotropein dramatically reduced hemorrhage and exhibited decreased loose, bloody, and DAI scores (Figure 1D). In the long-term intake of DSS, the DSS-treated group showed severe colon atrophy, congestion, and edema, in which the length of the colon was significantly shorter, and the thickness of the colon significantly increased compared to that of the NC group (Figures 1E–G). However, 5-ASA or monotropein dramatically alleviated colon shortening and thickening. In chronic colitis mice, the arrangement of colon epithelial cells was disordered, and their morphology was changed. The recess structure was destroyed, resulting in the loss of colon structure. These changes led to an increased histopathological score (Figure 1H). Treatment with 5-ASA or monotropein markedly improved colonic injury, as evidenced by an increased number of normal epithelial cells and intact crypts (Figure 1H). These results suggest that monotropein can potentially alleviate colonic injury in mice with chronic colitis.

### 3.2 Monotropein could inhibit the secretion of inflammatory cytokines and reduce intestinal permeability in chronic colitis mice

**FIGURE 2:**
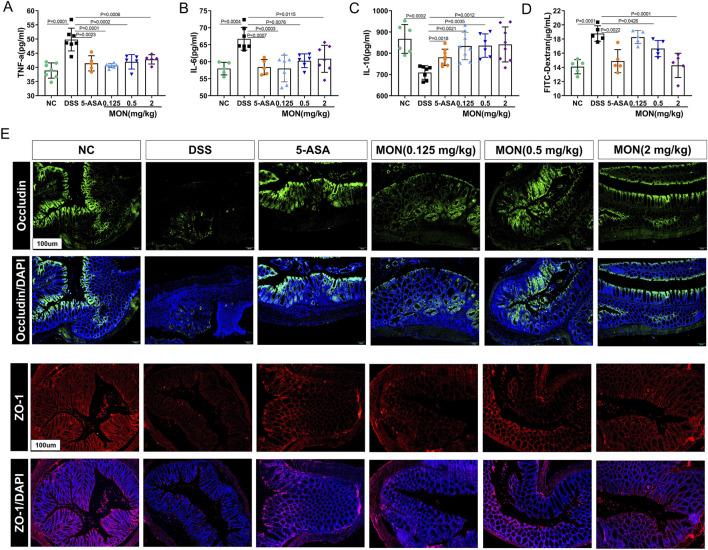
Monotropein inhibits the secretion of inflammatory cytokines and reduces intestinal permeability in mice with chronic colitis. **(A)** TNF-a level in serum. **(B)** IL-6 level in serum. **(C)** IL-10 level in serum. **(D)** Content of FITC-dextran in serum. **(E)** Representative images of occluding and ZO-1 protein positive expression (*n* = 3). The results are presented as mean ± SD (*n* = 8). Compared to the control group, ^#^ *P* < 0.05, ^##^ *P* < 0.01. Compared to the model group, ^*^ *P* < 0.05, ^**^ *P* < 0.01.

To investigate the impact of monotropein on modulating chronic colitis in mice, we assayed the serum levels of inflammatory factors in each group. Results indicated that the concentrations of TNF-a and IL-6 in the DSS-treated group significantly increased, and the concentration of IL-10 significantly decreased compared to those in the control group (Figures 2A–C). Conversely, both 5-ASA and monotropein could substantially decrease the expression of TNF-α and IL-6 and increase the expression of IL-10 in mice with chronic colitis (Figures 2A–C). Furthermore, we assessed the effect of monotropein on intestinal permeability in mice with chronic colitis. As illustrated in Figure 2D, compared to the control group, the levels of serum FITC-dextran were significantly increased in DSS-induced chronic colitis mice. Immunofluorescence results showed that the expressions of occludin and ZO-1 in the DSS-treated group were markedly reduced compared to that in the control group (Figure 2E). Monotropein treatment in chronic colitis mice could increase the expression of occludin and ZO-1 in a dose-dependent manner (Figure 2E). These findings suggested that monotropein could ameliorate inflammation and decrease intestinal permeability in mice with chronic colitis.

### 3.3 Monotropein could inhibit the intestinal fibers and epithelial–mesenchymal transition of colonic tissue in chronic colitis

**FIGURE 3:**
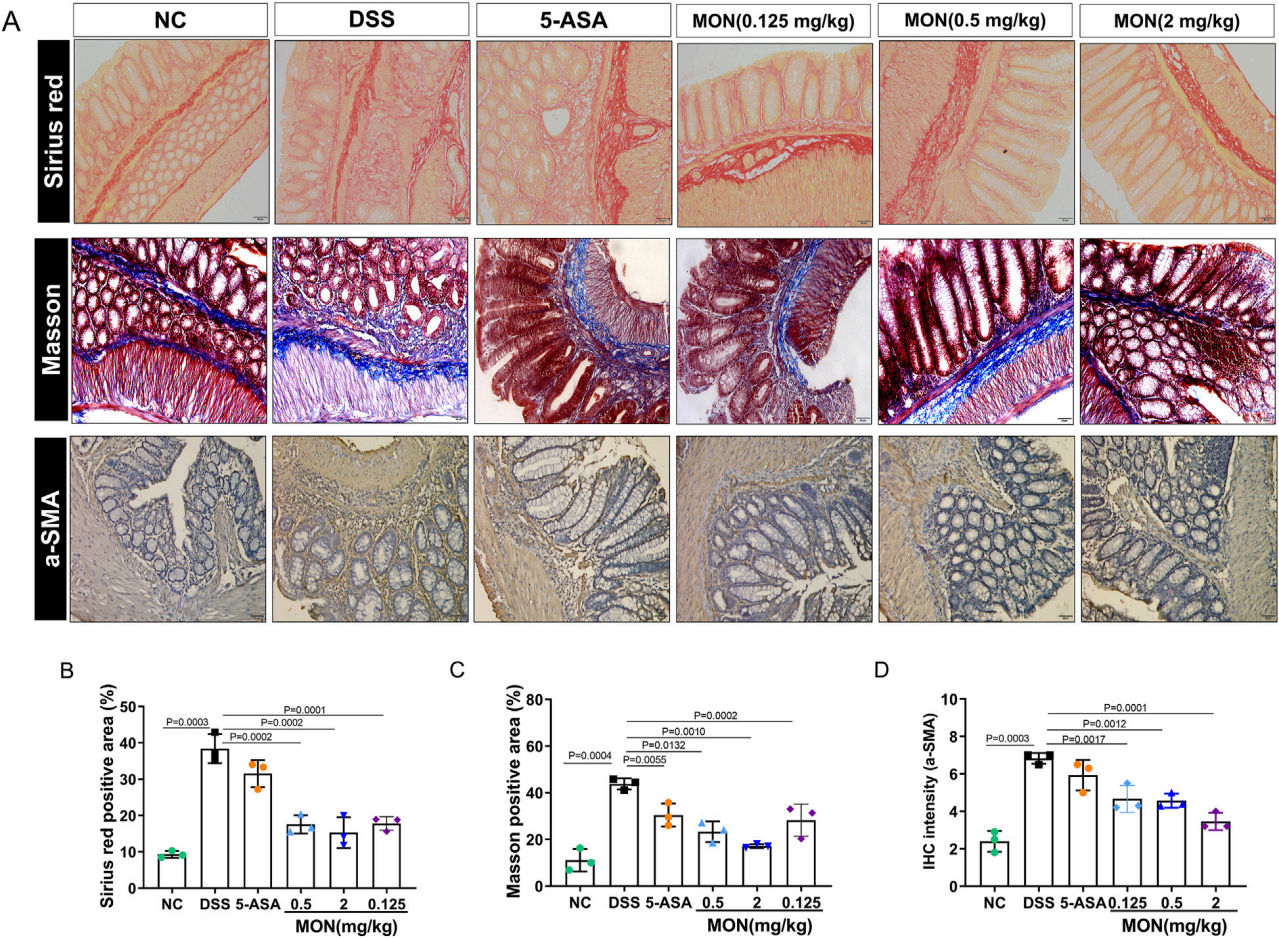
Monotropein inhibits the intestinal fibers and epithelial–mesenchymal transition of colonic tissue in chronic colitis. **(A)** Representative images of colon tissue with Sirius red, Masson, and a-SMA protein IHC staining. **(B)** Sirius red positive area (%). **(C)** Masson positive area (%). **(D)** IHC intensity of a-SMA protein. The results are presented as mean ± SD (*n* = 3). Compared to the control group, ^#^ *P* < 0.05, ^##^ *P* < 0.01. Compared to the model group, ^*^ *P* < 0.05, ^**^ *P* < 0.01.

Chronic inflammation of the intestine is characterized by repetitive damage and subsequent repair of intestinal tissue, ultimately resulting in the initiation and progression of intestinal fibrosis. Sirius red staining and Masson staining were performed on colon tissue to study the degree of intestinal fibrosis in each group of mice. The findings revealed that the colon tissue of mice exposed to DSS for an extended period exhibited a notable increase in fiber deposition. Among all groups, the DSS-treated group displayed the most severe symptoms (Figures 3A–C). Compared to the DSS-treated group, the fiber deposition of colon tissue in the 5-ASA or monotropein treatment groups improved to different degrees (Figures 3A–C). Immunohistochemical methods were used to analyze the signature proteins of the epithelial–mesenchymal transition. The results indicated that, compared to the control group, the DSS group exhibited a significant increase in the positive expression of α-smooth muscle actin (α-SMA) protein (Figures 3A, D). Compared to the DSS group, the positive expression of a-SMA protein in the monotropein treatment groups decreased to different degrees (Figures 3A, D). These findings imply that monotropein mitigates intestinal fibrosis and epithelial–mesenchymal transition in mice with chronic colitis.

### 3.4 Monotropein could regulate the mTOR/P70S6K pathway in chronic colitis

**FIGURE 4:**
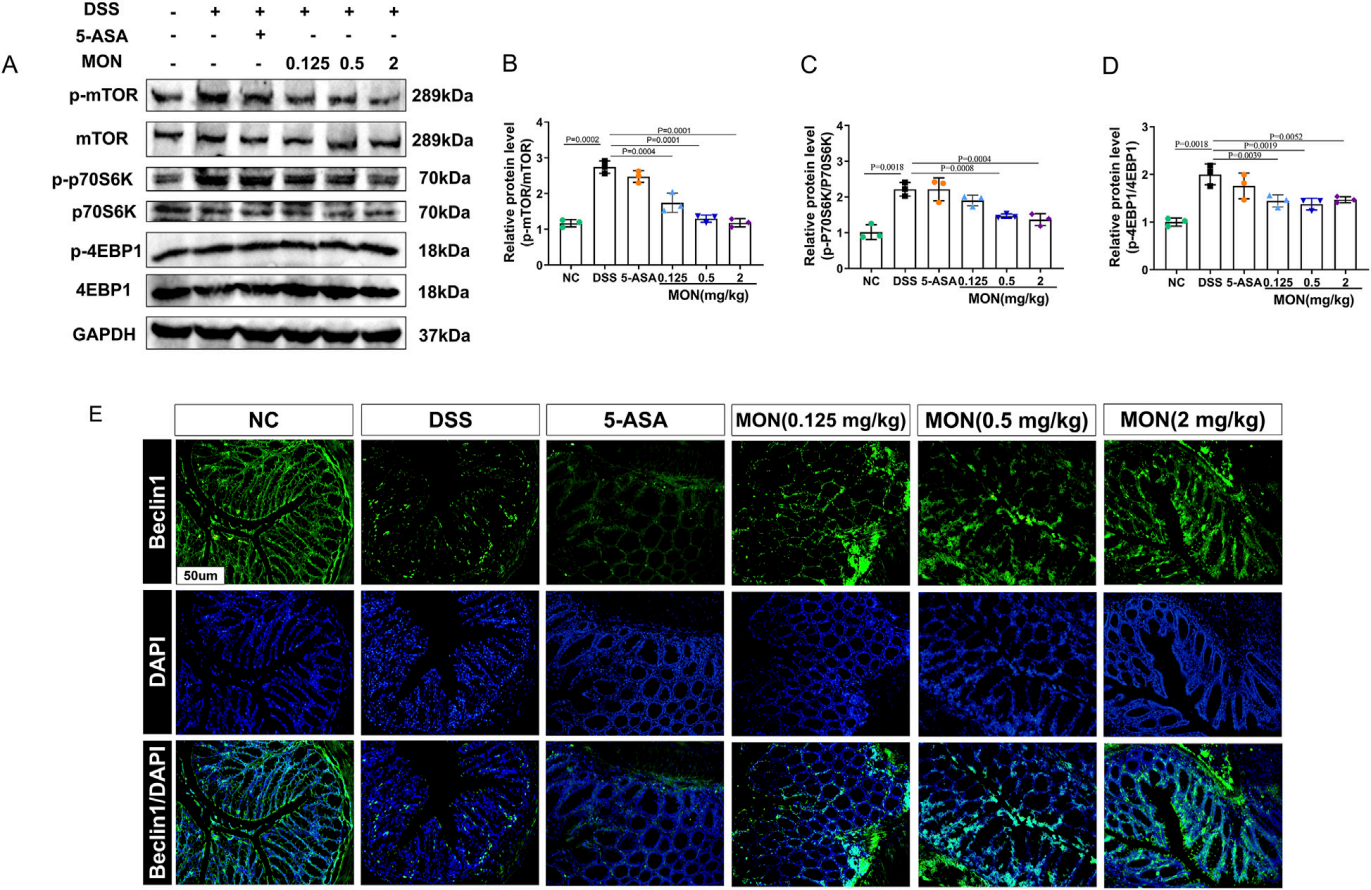
Monotropein regulates the mTOR/P70S6K pathway in chronic colitis. **(A)** Representative blot of each protein. **(B)** Relative protein level of p-mTOR/mTOR. **(C)** Relative protein level of p-P70S6K/P70S6K. **(D)** Relative protein level of p-4EBP1/4EBP1. **(E)** Representative images of Beclin1 protein positive expression. The results are presented as mean ± SD (*n* = 3). Compared to the control group, ^#^ *P* < 0.05, ^##^ *P* < 0.01. Compared to the model group, ^*^ *P* < 0.05, ^**^ *P* < 0.01.

To elucidate the mechanism through which monotropein inhibits epithelial–mesenchymal transition, we assessed the expression of proteins within the mTOR/P70S6K pathway, as well as the autophagy-associated protein Beclin1. Results showed that the phosphorylation of mTOR and its downstream proteins P70S6K and 4EBP1 were markedly increased in the DSS-treated group (Figures 4A–D). After monotropein treatment, the ratios of p-mTOR/mTOR, p-P70S6K/P70S6K, and p-4EBP1/4EBP1 significantly decreased (Figures 6A–D). Immunofluorescence results showed that the expression of autophagy-associated protein Beclin1 significantly decreased in the DSS-treated group, while monotropein treatment led to an increase in Beclin1 expression (Figure 4E). It should be noted that 5-ASA did not significantly regulate the expression of mTOR/P70S6K pathway proteins and Beclin1 (Figures 4A–E). These findings suggest that monotropein may inhibit epithelial–mesenchymal transition in chronic colitis by modulating autophagy through the mTOR/P70S6K pathway.

### 3.5 Monotropein could inhibit the epithelial–mesenchymal transition in TGF-β1-induced IEC-6 cells

**FIGURE 5:**
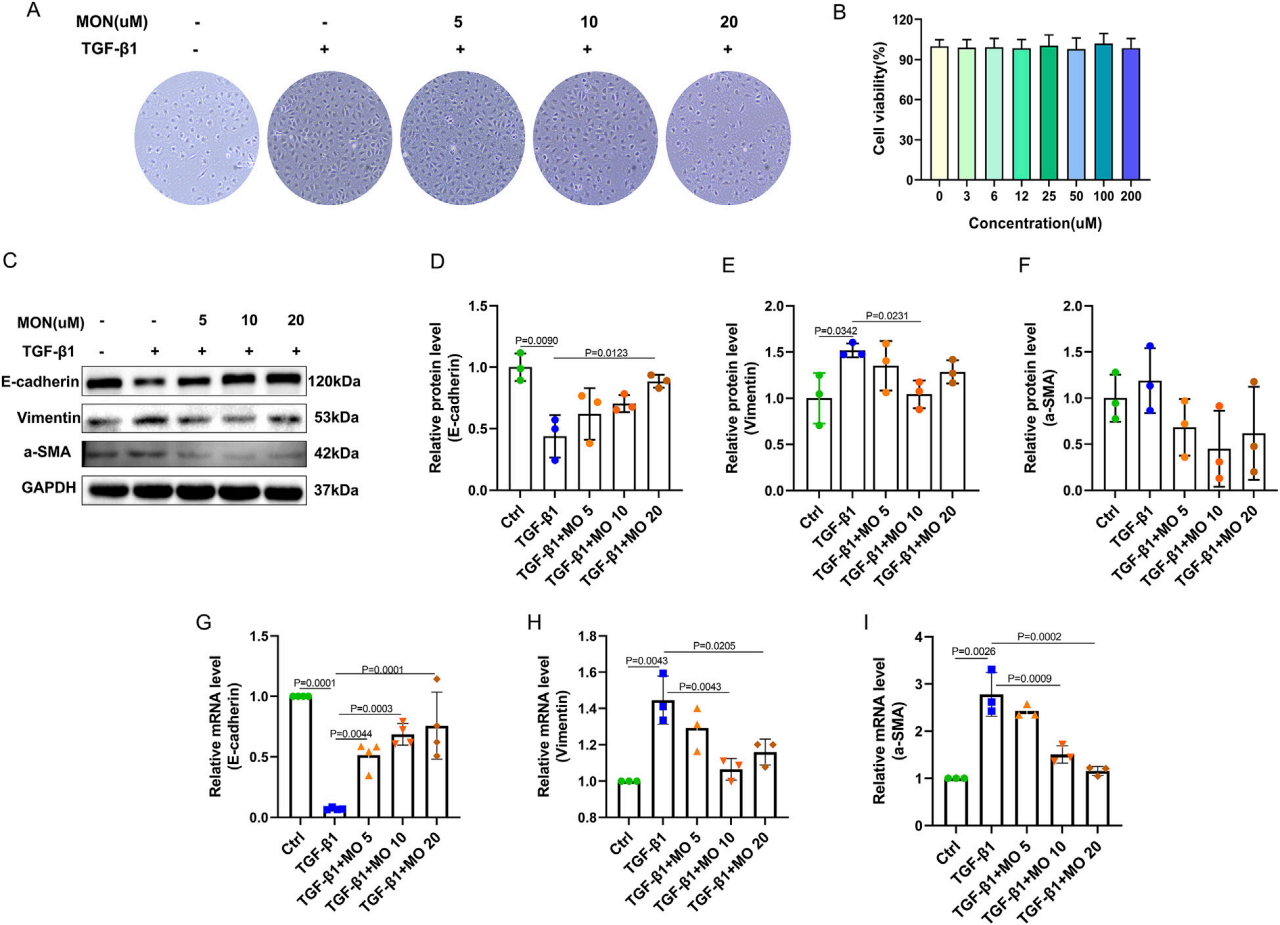
Monotropein inhibits the epithelial–mesenchymal transition in TGF-β1-induced IEC-6 cells. **(A)** Representative images of IEC-6 cells of each group. **(B)** CCK-8 analysis of the effect of different concentrations of monotropein on IEC-6 cell activity. **(C)** Representative blots of each protein. **(D)** Relative protein level of E-cadherin. **(E)** Relative protein level of Vimentin. **(F)** Relative protein level of a-SMA. **(G)** Relative mRNA level of E-cadherin. **(H)** Relative mRNA level of Vimentin. **(I)** Relative mRNA level of a-SMA. The results are presented as mean ± SD (*n* = 3). Compared to the control group, ^#^ *P* < 0.05, ^##^ *P* < 0.01. Compared to the model group, ^*^ *P* < 0.05, ^**^ *P* < 0.01.

We investigated the inhibitory effect of monotropein on epithelial–mesenchymal transition (EMT) *in vitro*, as well as the cytotoxicity of the drug on IEC-6 cells. Throughout the modeling process, the growth rate of IEC-6 cells accelerated, and they progressively adopted a fibroblast-like morphology (Figure 5A). The results demonstrated that TGF-β1-induced IEC-6 cells lost their epithelial phenotype and expressed a mesenchymal cell phenotype. Monotropein mitigated the loss of the epithelial phenotype in TGF-β1-induced IEC-6 cells (Figure 5A). The results indicated that TGF-β1-induced IEC-6 cells lost the epithelial phenotype and expressed a mesenchymal cell phenotype. Monotropein improved the loss of the epithelial phenotype in TGF-β1-induced IEC-6 cells (Figure 5A).

The cell viability test results showed that there was no cytotoxicity for monotropein to IEC-6 cells at any concentration up to 200 µmol/L (Figure 5B). Accordingly, the relative protein levels of E-cadherin markedly decreased, and the relative protein levels of vimentin and a-SMA increased (Figures 5C–F). The relative mRNA levels of E-cadherin significantly reduced, and the relative mRNA levels of Vimentin and a-SMA remarkably increased in the TGF-β1 group (Figures 5G–I). Monotropein could significantly upregulate the levels of E-cadherin mRNA and downregulate the levels of vimentin and a-SMA mRNA (Figures 5G–I). Consequently, it markedly increased the expression in the α-SMA protein (Figures 5C–F). Corresponding to the effects observed *in vivo*, these results revealed that monotropein could also effectively inhibit EMT *in vitro*.

### 3.6 Monotropein is directly bound to mTOR

**FIGURE 6:**
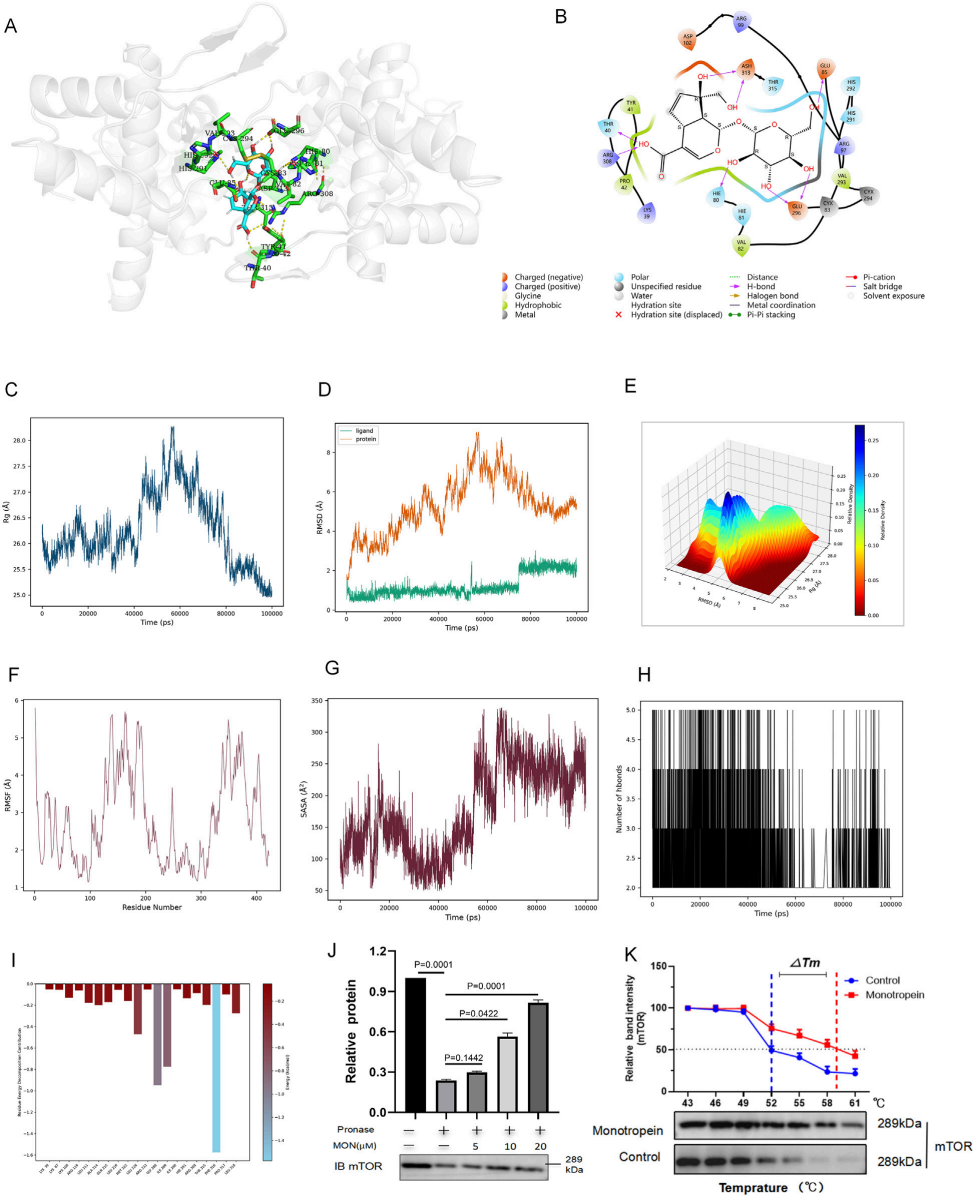
The molecular docking study and MD simulations between monotropein (MON) and mTOR. **(A)** The putative docking models between MON and mTOR. **(B)** 2D diagram of the interaction between MON and mTOR showing the major binding sites and bonding forces. **(C–H)** Rg, RMSD, RMSF, SASA, and H-bond number of mTOR-MON complex in MD simulations. **(I)** The energy contributions of each residue in the mTOR-MON complex. **(J)** mTOR expression levels with varying ratios of pronase and mTOR concentrations (1:100 pronase) assessed by WB. **(K)** mTOR expression levels with different temperatures and MON concentrations.

Molecular docking was conducted using AutoDock to explore the interaction between MON and mTOR. The results revealed that the mTOR-MON complex exhibited a robust binding affinity, with an average binding energy of −7.24 ± 0.12 kcal/mol (Figures 6A, B). To evaluate the binding stability of mTOR and MON, 100-ns MD simulations were executed on the docking-optimized mTOR-MON complex. The radius of gyration (Rg) affirmed the tightness of the complex, remaining relatively constant throughout the 100-ns simulations, with minor fluctuations observed between 50 and 80 ns (Figure 6C).

The overall conformational change was quantified by the root mean square deviation (RMSD). Lower RMSD values suggest a more stable protein–ligand complex. Figures 6D, E show that the RMSD of AS ranged between 1.50 Å and 2.00 Å throughout the simulation, while the RMSD of the protein varied between 2.50 Å and 8.00 Å. The relatively minor RMSD fluctuations during the MD simulations indicated the reliability of the docking poses (Figures 6D, E). Root mean square fluctuation (RMSF) analysis demonstrated that most protein residues remained stable (Figure 6F).

Solvent-accessible surface area (SASA) characterized the accessible surface area of the protein solvent that could be directly contacted over time (Figure 6G). Hydrogen bonding also plays a pivotal role in stabilizing protein–ligand interactions. During MD simulations, MON and mTOR formed stable hydrogen bonds, with the number of hydrogen bonds fluctuating between two and five, averaging four within 100 ns (Figure 6H), further validating the stability of the mTOR-MON complex. To pinpoint the key amino acid residues involved in mTOR protein binding, energetic decomposition of amino acid residues was performed. Figure 6I displayed the amino acid residues with energy contribution values below −1 kcal/mol, highlighting PHE316 as the primary contributor to binding free energy (Figure 6I).

The binding of drugs to target proteins induces conformational changes that alter the proteolytic and thermal stability of the target proteins. To further confirm the interaction potential between MON and mTOR, we used the DARTS and CETSA assays. The DARTS assay showed that mTOR undergoes approximately 70% proteolytic hydrolysis when MON is exposed to a pronase-to-total-protein ratio of 1:100. MON inhibited the proteolytic breakdown of mTOR in a dose-dependent manner until MON reached 20 μM (Figure 6J). CETSA results showed that MON significantly enhanced the thermal stability of mTOR under different temperature changes in a dose-dependent manner compared with the control. MON incubation also resulted in a significant increase in the thermal stability of mTOR compared with the control (Figure 6K).

### 3.7 Monotropein could regulate autophagy via the mTOR/P70S6K pathway in TGF-β1-induced IEC-6 cells to inhibit EMT

**FIGURE 7:**
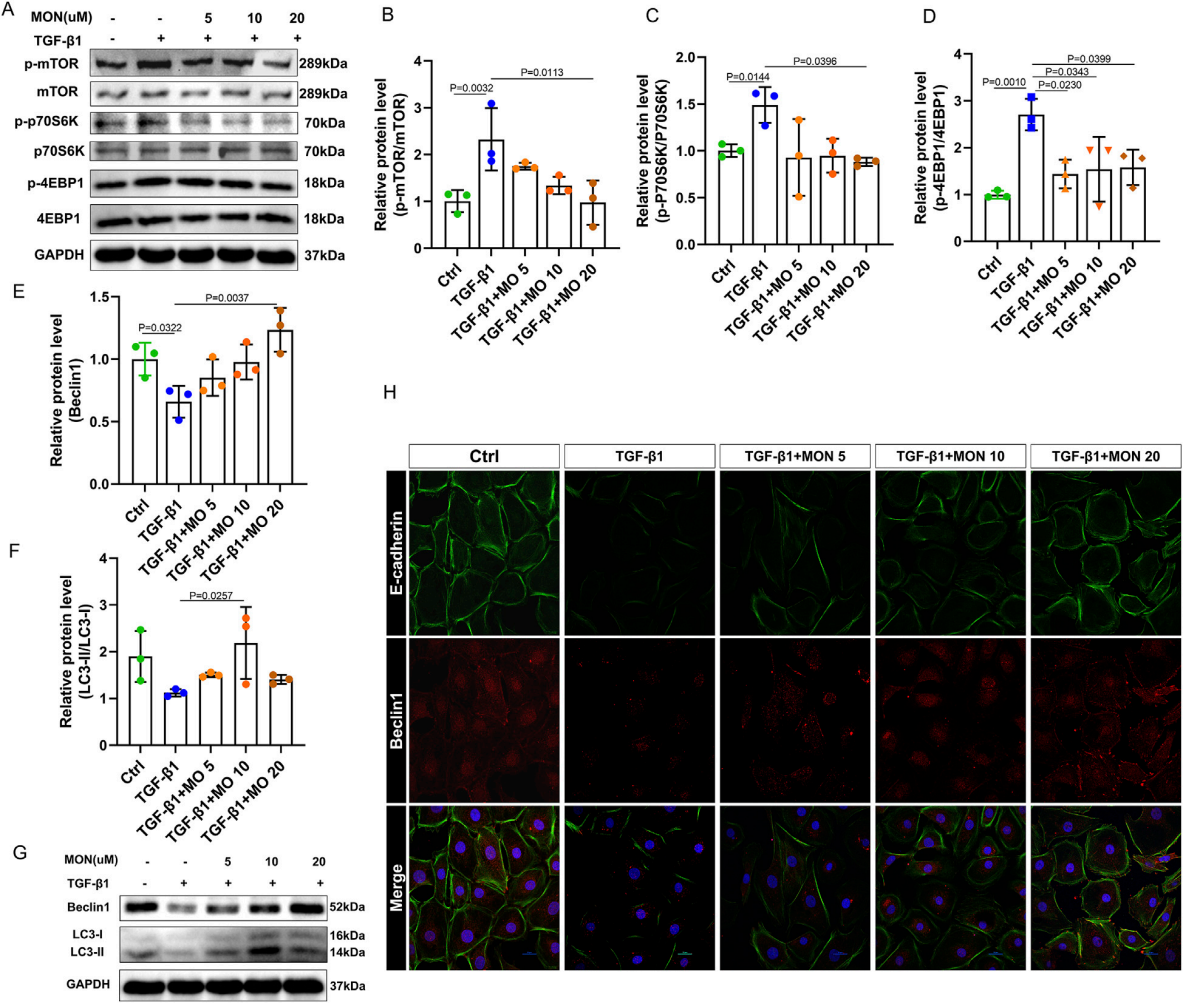
Monotropein regulates autophagy via the mTOR/P70S6K pathway in TGF-β1-induced IEC-6 cells to inhibit EMT. **(A)** Representative blots of each protein. **(B)** Relative protein level of p-mTOR/mTOR. **(C)** Relative protein level of p-P70S6K/P70S6K. **(D)** Relative protein level of p-4EBP1/4EBP1. **(E)** Relative protein level of Beclin1. **(F)** Relative protein level of LC-II/LC-I. **(G)** Representative blot of Beclin1 and LC3. **(H)** Representative images of cellular co-localization fluorescence of Beclin1 and E-cadherin proteins. The results are presented as mean ± SD (*n* = 3). Compared to the control group, ^#^ *P* < 0.05, ^##^ *P* < 0.01. Compared to the model group, ^*^ *P* < 0.05, ^**^ *P* < 0.01.

We evaluated the expression of the mTOR/P70S6K pathway and autophagy-associated proteins and genes, and the relationship between inhibition of EMT and regulation of autophagy was detected by immunofluorescence. Our findings indicated that TGF-β1 significantly induced the phosphorylation of mTOR and upregulated the ratios of p-p70S6K/p70S6K and p-4EBP1/4EBP1, effects that were reversed by monotropein (Figures 7A–D). Accordingly, the protein expression of autophagy marker genes BECN1 and LC3 remarkably decreased (Figures 7E–G). Conversely, monotropein significantly increased their protein expression compared to the TGF-β1 group (Figures 7E–G). Immunofluorescence analysis revealed that in the TGF-β1 group, the expression of E-cadherin protein was diminished, and Beclin1 protein expression was also reduced (Figure 7H). After monotropein administration, the expressions of E-cadherin and Beclin1 protein increased in a dose-dependent manner (Figure 7H). Collectively, these results imply that TGF-β1 exposure suppressed autophagy in IEC-6 cells and that monotropein can counteract this suppression to inhibit EMT.

## 4 Discussion

Chronic colitis is a recurrent condition characterized by multiple intestinal inflammatory edemas, ulceration, and bleeding lesions. The chronic colitis model reflects the dynamic process of development (Gupta et al., 2021), which is characterized by repeated damage and repair of intestinal tissue. This process leads to the occurrence of intestinal EMT, which further leads to the initiation and development of intestinal fibrosis. Inhibition of EMT to prevent colitis from transforming into fibrosis is key to treating IBD and improving the prognosis of IBD. The present study aimed to investigate the inhibitory effect of monotropein on intestinal EMT in a DSS-induced chronic colitis model in mice and further explore its underlying mechanism. The results provide substantial support for our hypotheses and yield significant implications for understanding the mechanisms of IBD and EMT.

The DSS-induced chronic colitis mice model has clinical features similar to colitis in humans, such as weight loss, loose stools, and bloody stools (Rogler, 2014). These key indexes were used to evaluate the effects of monotropein on the chronic colitis mouse model. The results demonstrated that monotropein treatment significantly inhibited weight loss, relieved loose stool and bloody stool symptoms, and improved the overall health status of the mice. These findings suggest that monotropein has a therapeutic effect on chronic colitis, which is in line with our hypothesis that it can ameliorate IBD symptoms.

Long-term ingestion of DSS can generate chemical damage to the intestinal epithelium, leading to colon atrophy and inflammation, disrupting the normal structure of the colon (Sun et al., 2020). Monotropein treatment of chronic colitis mice significantly inhibited colonic atrophy, relieved colonic congestion and edema, and reduced the damage to colonic structure. The therapeutic effect was similar to that of 5-ASA.

Inflammation is closely associated with the progression of colitis. An imbalance of pro-inflammatory and anti-inflammatory cytokines can prevent the recovery of inflammation and damage the mucosal tissue of the colon (Brudek, 2019; Rathinam and Chan, 2018). In this study, we found that 5-ASA or monotropein remarkably decreased serum levels of TNF-a and IL-6 cytokines and increased levels of anti-inflammatory cytokine IL-10 in chronic colitis mice. Intestinal permeability is an important indicator of intestinal barrier function. Monotropein can improve the distribution and diffusion of FITC-dextran in the intestinal tract of chronic colitis mice. Intestinal tight junction proteins, such as occludin and ZO-1, are essential mechanical barrier molecules to maintain intestinal mucosal integrity and regulate intestinal permeability, which are the first protective layer against the occurrence and development of colitis (Chen Y. et al., 2021; Shin and Kim, 2022). We found that after the treatment of monotropein, the tight junction proteins occludin and ZO-1 in the intestinal tract of chronic colitis mice were significantly upregulated. Taken together, these results suggested that monotropein could alleviate the structural damage of the colon, inhibit chronic colitis, and improve clinical symptoms of chronic colitis by inhibiting inflammation, remodeling the colon mucosal barrier and other mechanisms, thus becoming a potential therapeutic drug for chronic colitis.

The transformation of chronic colitis through EMT to fibrosis is an inevitable and serious consequence of the long-term progression of IBD (Barreiro-de Acosta et al., 2023). In this process, the expression of EMT markers such as the cell adhesion molecule E-cadherin decreases while the expression of the mesenchymal marker a-SMA increases, and the cytoskeleton of epithelial cells undergoes remodeling (Wang et al., 2021; Prados et al., 2021). In addition, pathological collagen fiber deposition in colon tissue is a significant sign of intestinal fibrosis (Wang et al., 2022). Our study found that monotropein treatment significantly reduced the deposition of pathological collagen fibers in colon tissue, as evidenced by Sirius red and Masson staining results. Furthermore, immunohistochemical analysis revealed a decrease in the positive expression of α-SMA protein in the monotropein groups compared to the DSS group. These results strongly suggest that monotropein can inhibit the EMT process in chronic colitis, thereby potentially preventing the progression to fibrosis.

To further validate our findings, we conducted *in vitro* experiments using IEC-6 cells induced with TGF-β1 to establish a fibrosis model. The results showed that monotropein improved the loss of epithelial phenotype in TGF-β1-induced IEC-6 cells and inhibited the expression of mesenchymal markers such as Vimentin and α-SMA. These *in vitro* findings are consistent with our *in vivo* results and support our hypothesis that monotropein inhibits EMT.

We further verified the inhibitory effect of monotropein on EMT *in vitro*, although the mechanism by which monotropein inhibits EMT is unknown. In recent years, many studies have shown the regulatory effect of autophagy on EMT (Gundamaraju et al., 2022a; Babaei et al., 2021) and that the increase of autophagy activity can inhibit EMT (Kong et al., 2020). In our study, we observed that monotropein treatment inhibited the phosphorylation of mTOR and its downstream proteins P70S6K and 4EBP1, which are known to negatively regulate autophagy. Concurrently, there was an increase in autophagy activity, as evidenced by the increased expression of the autophagy-associated protein Beclin1. These results suggest that monotropein might inhibit EMT in chronic colitis by regulating autophagy via the mTOR/P70S6K pathway, which is in accordance with our study hypothesis.

Molecular docking studies and MD simulations revealed a strong binding affinity between monotropein and mTOR, with an average binding energy of −7.24 ± 0.12 kcal/mol. This direct interaction between monotropein and mTOR provides additional evidence for its mechanism of action in inhibiting the mTOR/P70S6K pathway and regulating autophagy. The increased thermal stability of mTOR in the presence of monotropein, as shown by CETSA results, further supports this notion.

In conclusion, the results of our study demonstrate that monotropein has a therapeutic effect on chronic colitis and can inhibit the transformation of chronic colitis into fibrosis by regulating autophagy via the mTOR/P70S6K pathway. This study contributes to a deeper understanding of the mechanisms of IBD and EMT and identifies monotropein as a potential drug for the treatment of chronic colitis and the prevention of intestinal fibrosis. These findings have significant implications for the development and application of monotropein in alleviating IBD and preventing colitis from transforming into fibrosis.

## 5 Conclusion

This study identified monotropein as a potential drug for the treatment of chronic colitis and the prevention of intestinal fibrosis. In addition to improving colon inflammation, monotropein inhibits the development of EMT by at least partially regulating autophagy via the mTOR/P70S6K pathway.

## Data Availability

The original contributions presented in the study are included in the article/Supplementary Material; further inquiries can be directed to the corresponding authors.

## Abbreviations

5-ASA, 5-aminosalicylic acid; a-SMA, a-smooth muscle actin; DSS, dextran sodium sulfate; DAI, disease activity index; EMT, epithelial–mesenchymal transition; 4EBP1, eIF4E-binding protein 1; FITC-dextran, fluorescein isothiocyanate dextran; H&E, hematoxylin–eosin; IBD, inflammatory bowel disease; IL-6, interleukin-6; IL-10, interleukin-10; IHC, immunohistochemistry; MON, monotropein; mTOR, mammalian target of rapamycin; P70S6K, ribosomal protein S6 kinase beta-1; TNF-a, tumor necrosis factor-a; TGF-β1, transforming growth factor-β1; ZO-1, zona occludens 1.

## References

[B1] Babaei G. Aziz S. G. Jaghi N. Z. Z. (2021). EMT, cancer stem cells and autophagy; the three main axes of metastasis. Biomed. Pharmacother. 133, 110909. 10.1016/j.biopha.2020.110909 33227701

[B2] Barreiro-de Acosta M. Molero A. Artime E. Diaz-Cerezo S. Lizan L. de Paz H. D. (2023). Epidemiological, clinical, patient-reported and economic burden of inflammatory bowel disease (ulcerative colitis and Crohn’s disease) in Spain: a systematic review. Adv. Ther. 40, 1975–2014. 10.1007/s12325-023-02473-6 36928496 PMC10129998

[B3] Brudek T. (2019). Inflammatory bowel diseases and Parkinson's disease. J. Park. Dis. 9, S331–S344. 10.3233/JPD-191729 PMC6839501 31609699

[B4] Chen Y. Chen H. Ding J. Stanton C. Ross R. P. Zhao J. (2021b). *Bifidobacterium longum* ameliorates dextran sulfate sodium-induced colitis by producing conjugated linoleic acid, protecting intestinal mechanical barrier, restoring unbalanced gut microbiota, and regulating the toll-like receptor-4/nuclear factor-κB signaling pathway. J. Agric. Food Chem. 69, 14593–14608. 10.1021/acs.jafc.1c06176 34843239

[B5] Chen Y. Liang J. Chen S. Lin N. Xu S. Miao J. (2024). Discovery of vitexin as a novel VDR agonist that mitigates the transition from chronic intestinal inflammation to colorectal cancer. Mol. Cancer 23, 196. 10.1186/s12943-024-02108-6 39272040 PMC11395219

[B6] Chen Y. Lu Y. Pei C. Liang J. Ding P. Chen S. (2020). Monotropein alleviates secondary liver injury in chronic colitis by regulating TLR4/NF-κB signaling and NLRP3 inflammasome. Eur. J. Pharmacol. 883, 173358. 10.1016/j.ejphar.2020.173358 32710952

[B7] Chen Y. Zhu S. Chen Z. Liu Y. Pei C. Huang H. (2022). Gingerenone A alleviates ferroptosis in secondary liver injury in colitis mice via activating Nrf2-Gpx4 signaling pathway. J. Agric. Food Chem. 70, 12525–12534. 10.1021/acs.jafc.2c05262 36135333

[B8] Chen Y.-e. Xu S.-j. Lu Y.-y. Chen S.-x. Du X.-h. Hou S.-z. (2021a). Asperuloside suppressing oxidative stress and inflammation in DSS-induced chronic colitis and RAW 264.7 macrophages via Nrf2/HO-1 and NF-κB pathways. Chemico-Biological Interact. 344, 109512. 10.1016/j.cbi.2021.109512 33974900

[B9] Dominguez C. David J. M. Palena C. (2017). Epithelial-mesenchymal transition and inflammation at the site of the primary tumor. Semin. Cancer Biol. 47, 177–184. 10.1016/j.semcancer.2017.08.002 28823497 PMC5698091

[B10] Etemadi A. Safiri S. Sepanlou S. G. Ikuta K. Bisignano C. Shakeri R. (2020). The global, regional, and national burden of stomach cancer in 195 countries, 1990-2017: a systematic analysis for the Global Burden of Disease study 2017. Lancet Gastroenterology and Hepatology 5, 42–54. 10.1016/S2468-1253(19)30328-0 31648970 PMC7033564

[B11] Flier S. N. Tanjore H. Kokkotou E. G. Sugimoto H. Zeisberg M. Kalluri R. (2010). Identification of epithelial to mesenchymal transition as a novel source of fibroblasts in intestinal fibrosis. J. Biol. Chem. 285, 20202–20212. 10.1074/jbc.M110.102012 20363741 PMC2888433

[B12] Funakoshi T. Yamashita K. Ichikawa N. Fukai M. Suzuki T. Goto R. (2012). A novel NF-κB inhibitor, dehydroxymethylepoxyquinomicin, ameliorates inflammatory colonic injury in mice. J. Crohns Colitis 6, 215–225. 10.1016/j.crohns.2011.08.011 22325176

[B13] Gellerstedt M. (2002). Statistical issues - significantly important in medical research. Allergy 57, 76–82. 10.1034/j.1398-9995.2002.1r151.x 11929408

[B14] Grassi G. Di Caprio G. Santangelo L. Fimia G. M. Cozzolino A. M. Komatsu M. (2015). Autophagy regulates hepatocyte identity and epithelial-to-mesenchymal and mesenchymal-to-epithelial transitions promoting Snail degradation. Cell Death Dis. 6, e1880. 10.1038/cddis.2015.249 26355343 PMC4650445

[B15] Gundamaraju R. Lu W. Paul M. K. Jha N. K. Gupta P. K. Ojha S. (2022a). Autophagy and EMT in cancer and metastasis: who controls whom? Biochim. Biophys. Acta Mol. Basis Dis. 1868, 166431. 10.1016/j.bbadis.2022.166431 35533903

[B16] Gundamaraju R. Lu W. Y. Paul M. K. Jha N. K. Gupta P. K. Ojha S. (2022b). Autophagy and EMT in cancer and metastasis: who controls whom? Biochimica Biophysica Acta-Molecular Basis Dis. 1868, 166431. 10.1016/j.bbadis.2022.166431 35533903

[B17] Gupta V. Mohsen W. Chapman T. P. Satsangi J. (2021). Predicting outcome in acute severe colitis-controversies in clinical practice in 2021. J. Crohns Colitis 15, 1211–1221. 10.1093/ecco-jcc/jjaa265 33388777 PMC7799290

[B18] Jo Y. K. Roh S. A. Lee H. Park N. Y. Choi E. S. Oh J. H. (2017). Polypyrimidine tract-binding protein 1-mediated down-regulation of ATG10 facilitates metastasis of colorectal cancer cells. Cancer Lett. 385, 21–27. 10.1016/j.canlet.2016.11.002 27836735

[B19] Kong D. Zhang Z. Chen L. Huang W. Zhang F. Wang L. (2020). Curcumin blunts epithelial-mesenchymal transition of hepatocytes to alleviate hepatic fibrosis through regulating oxidative stress and autophagy. Redox Biol. 36, 101600. 10.1016/j.redox.2020.101600 32526690 PMC7287144

[B20] Lamouille S. Xu J. Derynck R. (2014). Molecular mechanisms of epithelial-mesenchymal transition. Nat. Rev. Mol. Cell Biol. 15, 178–196. 10.1038/nrm3758 24556840 PMC4240281

[B21] Liu J. Chen Y. Zhang J. Zheng Y. An Y. Xia C. (2024). Vitexin alleviates MNNG-induced chronic atrophic gastritis via inhibiting NLRP3 inflammasome. J. Ethnopharmacol. 340, 119272. 10.1016/j.jep.2024.119272 39716512

[B22] Lopez-Novoa J. M. Nieto M. A. (2009). Inflammation and EMT: an alliance towards organ fibrosis and cancer progression. EMBO Mol. Med. 1, 303–314. 10.1002/emmm.200900043 20049734 PMC3378143

[B23] Lu Y. Guan T. Xu S. Chen Y.-e. Shen Q. Zhu S. (2022). Asperuloside inhibited epithelial-mesenchymal transition in colitis associated cancer via activation of vitamin D receptor. Phytomedicine 101, 154070. 10.1016/j.phymed.2022.154070 35523114

[B24] Macias-Ceja D. C. Ortiz-Masia D. Salvador P. Gisbert-Ferrandiz L. Hernandez C. Hausmann M. (2019). Succinate receptor mediates intestinal inflammation and fibrosis. Mucosal Immunol. 12, 178–187. 10.1038/s41385-018-0087-3 30279517

[B25] Mathur R. Alam M. M. Zhao X. F. Liao Y. Shen J. Morgan S. (2019). Induction of autophagy in Cx3cr1(+) mononuclear cells limits IL-23/IL-22 axis-mediated intestinal fibrosis. Mucosal Immunol. 12, 612–623. 10.1038/s41385-019-0146-4 30765845 PMC6927046

[B26] Nieto M. A. Huang R. Y. Jackson R. A. Thiery J. P. (2016). Emt: 2016. Cell 166, 21–45. 10.1016/j.cell.2016.06.028 27368099

[B27] Prados M. E. Garcia-Martin A. Unciti-Broceta J. D. Palomares B. Collado J. A. Minassi A. (2021). Betulinic acid hydroxamate prevents colonic inflammation and fibrosis in murine models of inflammatory bowel disease. Acta Pharmacol. Sin. 42, 1124–1138. 10.1038/s41401-020-0497-0 32811965 PMC8209138

[B28] Rathinam V. A. K. Chan F. K. (2018). Inflammasome, inflammation, and tissue homeostasis. Trends Mol. Med. 24, 304–318. 10.1016/j.molmed.2018.01.004 29433944 PMC6456255

[B29] Rogler G. (2014). Chronic ulcerative colitis and colorectal cancer. Cancer Lett. 345, 235–241. 10.1016/j.canlet.2013.07.032 23941831

[B30] Scharl M. Huber N. Lang S. Furst A. Jehle E. Rogler G. (2015). Hallmarks of epithelial to mesenchymal transition are detectable in Crohn's disease associated intestinal fibrosis. Clin. Transl. Med. 4, 1. 10.1186/s40169-015-0046-5 25852817 PMC4384762

[B31] Sferra R. Pompili S. Ventura L. Dubuquoy C. Speca S. Gaudio E. (2018). Interaction between sphingosine kinase/sphingosine 1 phosphate and transforming growth factor-β/Smads pathways in experimental intestinal fibrosis. An *in vivo* immunohistochemical study. Eur. J. Histochem 62, 2956. 10.4081/ejh.2018.2956 30064196 PMC6077868

[B32] Shi Y. Li T. Zhou J. Li Y. Chen L. Shang H. (2019). Herbs-partitioned moxibustion combined with acupuncture inhibits TGF-β1-smad-snail-induced intestinal epithelial mesenchymal transition in Crohn's disease model rats. Evid. Based Complement. Altern. Med. 2019, 8320250. 10.1155/2019/8320250 PMC6582898 31275422

[B33] Shin J. S. Yun K. J. Chung K. S. Seo K. H. Park H. J. Cho Y. W. (2013a). Monotropein isolated from the roots of Morinda officinalis ameliorates proinflammatory mediators in RAW 264.7 macrophages and dextran sulfate sodium (DSS)-induced colitis via NF-κB inactivation. Food Chem. Toxicol. 53, 263–271. 10.1016/j.fct.2012.12.013 23261679

[B34] Shin J. S. Yun K. J. Chung K. S. Seo K. H. Park H. J. Cho Y. W. (2013b). Monotropein isolated from the roots of *Morinda officinalis* ameliorates proinflammatory mediators in RAW 264.7 macrophages and dextran sulfate sodium (DSS)-induced colitis via NF-κB inactivation. Food Chem. Toxicol. 53, 263–271. 10.1016/j.fct.2012.12.013 23261679

[B35] Shin W. Kim H. J. (2022). 3D *in vitro* morphogenesis of human intestinal epithelium in a gut-on-a-chip or a hybrid chip with a cell culture insert. Nat. Protoc. 17, 910–939. 10.1038/s41596-021-00674-3 35110737 PMC9675318

[B36] Solinas G. Marchesi F. Garlanda C. Mantovani A. Allavena P. (2010). Inflammation-mediated promotion of invasion and metastasis. Cancer Metastasis Rev. 29, 243–248. 10.1007/s10555-010-9227-2 20414701

[B37] Suarez-Carmona M. Lesage J. Cataldo D. Gilles C. (2017). EMT and inflammation: inseparable actors of cancer progression. Mol. Oncol. 11, 805–823. 10.1002/1878-0261.12095 28599100 PMC5496491

[B38] Sun M. Liu Y. Song Y. Gao Y. Zhao F. Luo Y. (2020). The ameliorative effect of Lactobacillus plantarum-12 on DSS-induced murine colitis. Food Funct. 11, 5205–5222. 10.1039/d0fo00007h 32458908

[B39] Tian X. Tian X. Huo R. Chang Q. Zheng G. Du Y. (2017). Bacillus Calmette-Guerin alleviates airway inflammation and remodeling by preventing TGF-β1 induced epithelial-mesenchymal transition. Hum. Vaccin Immunother. 13, 1758–1764. 10.1080/21645515.2017.1313366 28441064 PMC5557244

[B40] Torle J. Dabir P. D. Korsgaard U. Christiansen J. Qvist N. El-Hussuna A. (2020). Levels of intestinal inflammation and fibrosis in resection specimens after preoperative anti-tumor necrosis factor alpha treatment in patients with Crohn's disease: a comparative pilot study. Surg. Res. Pract. 2020, 6085678. 10.1155/2020/6085678 32149183 PMC7054778

[B41] Wang R. Wang D. Wang H. Wang T. Weng Y. Zhang Y. (2021). Therapeutic targeting of Nrf2 signaling by maggot extracts ameliorates inflammation-associated intestinal fibrosis in chronic DSS-induced colitis. Front. Immunol. 12, 670159. 10.3389/fimmu.2021.670159 34456904 PMC8387595

[B42] Wang Y. Huang B. Jin T. Ocansey D. K. W. Jiang J. Mao F. (2022). Intestinal fibrosis in inflammatory bowel disease and the prospects of mesenchymal stem cell therapy. Front. Immunol. 13, 835005. 10.3389/fimmu.2022.835005 35370998 PMC8971815

[B43] Xu S. Chen Y. Miao J. Li Y. Liu J. Zhang J. (2024). Esculin inhibits hepatic stellate cell activation and CCl4-induced liver fibrosis by activating the Nrf2/GPX4 signaling pathway. Phytomedicine 128, 155465. 10.1016/j.phymed.2024.155465 38471319

[B44] Yun S. M. Kim S. H. Kim E. H. (2019). The molecular mechanism of transforming growth factor-β signaling for intestinal fibrosis: a mini-review. Front. Pharmacol. 10, 162. 10.3389/fphar.2019.00162 30873033 PMC6400889

[B45] Zhang J. Liang F. Chen Z. Chen Y. Yuan J. Xiong Q. (2022). Vitexin protects against dextran sodium sulfate-induced colitis in mice and its potential mechanisms. J. Agric. Food Chem. 70, 12041–12054. 10.1021/acs.jafc.2c05177 36124900

[B46] Zhou X. Zhang K. Qi W. Zhou Y. Hong T. Xiong T. (2019). Exopolysaccharides from lactobacillus plantarum NCU116 enhances colonic mucosal homeostasis by controlling epithelial cell differentiation and c-Jun/Muc2 signaling. J. Agric. Food Chem. 67, 9831–9839. 10.1021/acs.jafc.9b03939 31407897

[B47] Zhu L. L. Du J. M. Dai Y. Y. Shen Y. Li H. M. Zhang Q. L. (2024). *Morinda officinalis* iridoid glycosides alleviate methotrexate-induced liver injury in CIA rats by increasing liver autophagy and improving lipid metabolism homeostasis. J. Ethnopharmacol. 333, 118486. 10.1016/j.jep.2024.118486 38914148

[B48] Zou X. Z. Liu T. Gong Z. C. Hu C. P. Zhang Z. (2017). MicroRNAs-mediated epithelial-mesenchymal transition in fibrotic diseases. Eur. J. Pharmacol. 796, 190–206. 10.1016/j.ejphar.2016.12.003 27916556

